# Supplementary material for: QTL analysis of Malus baccata ‘Jackii’-derived offspring reveals a polygenic inheritance pattern of apple blotch resistance
Source: Front Plant Sci. 2026 Jun 3;17:1854916. doi: 10.3389/fpls.2026.1854916 (PMC13272127; doi:10.3389/fpls.2026.1854916)
Supplement: Supplementary file 1 [file Table1.docx]

Supplementary Material

# Supplementary Tables

**Table S1.** List of SSR markers employed for genotyping genotypes derived from open pollination

| **Marker** | **Reference** |
| --- | --- |
| CH01f02, CH01f03b, CH01f07a, CH01h01, CH02c09, CH02d08, CH02g09, CH03d07, CH05e03, CH05f06 | Liebhard et al., 2002 |
| GD 12 | Hokanson et al., 1998 |

**Table S2**. Genome-wide significant associations identified by simple interval mapping in the detached leaf assay across all datasets

| **Trait** | **Year of inoculation** | **Inoculum** | **Leaf origin** | **Repetition** | **dpi** | **LG** | **Highest LOD score** | **Marker with highest LOD score** | **Position (cM)** | **Explained phenotypic variance (%)** |
| --- | --- | --- | --- | --- | --- | --- | --- | --- | --- | --- |
| **Necrotic spots per dm^2^** | **2023** | **2022** | **Field** | **1** | **7** | **8** | **7.37** | **HT1_LG08_25126438** | **51.2** | **24.3** |
| Necrotic spots per dm^2^ | 2023 | 2022 | Field | 1 | 10 | 8 | 5.46 | HT1_LG08_28047111 | 54.6 | 19.2 |
| **Necrotic spots per dm^2^** | **2023** | **2022** | **Field** | **1** | **14** | **9** | **4.49** | **HT1_LG09_6055205** | **33.0** | **16.4** |
| Necrotic spots per dm^2^ | 2023 | 2022 | Greenhouse | 2 | 10 | 3 | 3.30 | CH03e03 | 0.0 | 12.0 |
| **Necrotic spots per dm^2^** | **2023** | **2022** | **Greenhouse** | **2** | **14** | **3** | **4.86** | **CH03e03** | **0.0** | **17.2** |
| **Necrotic spots per dm^2^** | **2023** | **2022** | **Greenhouse** | **2** | **14** | **11** | **4.00** | **HT1_LG11_34294231** | **45.2** | **20.2** |
| **Necrotic spots per dm^2^** | **2023** | **2022** | **Greenhouse** | **1-3** | **7** | **5** | **4.95** | **HT1_LG05_24210086** | **19.0** | **21.5** |
| Necrotic spots per dm^2^ | 2023 | 2022 | Greenhouse | 1-3 | 10 | 5 | 4.06 | HT1_LG05_24210086 | 19.0 | 15.6 |
| Necrotic spots per dm^2^ | 2023 | 2022 | Greenhouse | 1-3 | 10 | 11 | 4.05 | HT1_LG11_34294231 | 45.2 | 20.9 |
| **Necrotic spots per dm^2^** | **2023** | **2022** | **Greenhouse** | **1-3** | **14** | **11** | **4.28** | **HT1_LG11_34294231** | **45.2** | **21.3** |
| **Necrotic spots per dm^2^** | **2025** | **2023** | **Field** | **4** | **14** | **15** | **4.93** | **HT1_LG15_41074458** | **79.2** | **17.4** |
| **AUDPC of necrotic spots per dm^2^** | **2023** | **2022** | **Field** | **1** | **7** | **8** | **7.37** | **HT1_LG08_25126438** | **51.2** | **24.3** |
| AUDPC of necrotic spots per dm^2^ | 2023 | 2022 | Field | 1 | 10 | 8 | 7.02 | HT1_LG08_25126438 | 51.2 | 23.4 |
| AUDPC of necrotic spots per dm^2^ | 2023 | 2022 | Field | 1 | 10 | 9 | 4.40 | HT1_LG09_5217202 | 32.0 | 16.3 |
| AUDPC of necrotic spots per dm^2^ | 2023 | 2022 | Field | 1 | 14 | 8 | 5.53 | HT1_LG08_28047111 | 54.6 | 19.3 |
| **AUDPC of necrotic spots per dm^2^** | **2023** | **2022** | **Field** | **1** | **14** | **9** | **4.45** | **HT1_LG09_6055205** | **33.0** | **16.5** |
| **AUDPC of necrotic spots per dm^2^** | **2023** | **2022** | **Field** | **1-3** | **10** | **8** | **4.70** | **HT1_LG08_25126438** | **51.2** | **16.3** |
| **AUDPC of necrotic spots per dm^2^** | **2023** | **2022** | **Greenhouse** | **2** | **10** | **5** | **4.16** | **HT1_LG05_24210086** | **19.0** | **16.5** |
| **AUDPC of necrotic spots per dm^2^** | **2023** | **2022** | **Greenhouse** | **2** | **14** | **3** | **3.82** | **CH03e03** | **0.0** | **13.8** |
| **AUDPC of necrotic spots per dm^2^** | **2023** | **2022** | **Greenhouse** | **3** | **10** | **5** | **5.00** | **HT1_LG05_6790436** | **3.7** | **17.8** |
| AUDPC of necrotic spots per dm^2^ | 2023 | 2022 | Greenhouse | 1-3 | 7 | 5 | 4.95 | HT1_LG05_24210086 | 19.0 | 21.5 |
| **AUDPC of necrotic spots per dm^2^** | **2023** | **2022** | **Greenhouse** | **1-3** | **10** | **5** | **6.04** | **HT1_LG05_24210086** | **19.0** | **25.0** |
| Percentage of necrotic leaf area | 2023 | 2022 | Greenhouse | 3 | 7 | 16 | 5.00 | HT1_LG16_16651738 | 57.5 | 23.8 |
| **Percentage of necrotic leaf area** | **2023** | **2022** | **Greenhouse** | **3** | **10** | **16** | **5.04** | **HT1_LG16_16651738** | **57.5** | **23.9** |
| Percentage of necrotic leaf area | 2023 | 2022 | Greenhouse | 3 | 14 | 16 | 4.61 | HT1_LG16_16651738 | 57.5 | 21.9 |
| **AUDPC of percentage of necrotic leaf area** | **2023** | **2022** | **Field** | **1-3** | **14** | **10** | **4.78** | **FRM4** | **27.2** | **18.0** |
| AUDPC of percentage of necrotic leaf area | 2023 | 2022 | Greenhouse | 3 | 7 | 16 | 5.00 | HT1_LG16_16651738 | 57.5 | 23.8 |
| AUDPC of percentage of necrotic leaf area | 2023 | 2022 | Greenhouse | 3 | 10 | 16 | 5.02 | HT1_LG16_16651738 | 57.5 | 23.9 |
| **AUDPC of percentage of necrotic leaf area** | **2023** | **2022** | **Greenhouse** | **3** | **14** | **16** | **5.11** | **HT1_LG16_16651738** | **57.5** | **24.2** |
| **Presence/absence of necrosis with acervuli surrounded by dark-brown tissue** | **2023** | **2022** | **Field** | **3** | **21** | **14** | **5.90** | **HT1_LG14_28796794** | **54.0** | **23.8** |

For each unique combination, the row corresponding to the marker with the highest logarithm of the odds (LOD) score per linkage group (LG) across all time points is highlighted in bold. AUDPC: area under the disease progress curve; cM: centimorgan; dpi: days post-inoculation.

**Table S3.** Genome-wide significant associations identified by simple interval mapping in the greenhouse experiment across all datasets

| **Trait** | **Year of inoculation** | **Inoculum** | **Treatment** | **Repetition** | **dpi** | **LG** | **Highest LOD score** | **Marker with highest LOD score*** | **Position (cM)** | **Explained phenotypic variance (%)** |
| --- | --- | --- | --- | --- | --- | --- | --- | --- | --- | --- |
| Phenotyping score | 2023 | 2022 | With zip bag | 1 | 28 | 14 | 4.39 | HT1_LG14_19111015 | 19.3 | 16.7 |
| Phenotyping score | 2023 | 2022 | With zip bag | 1 | 35 | 14 | 4.52 | HT1_LG14_27543259 | 49.2 | 16.4 |
| **Phenotyping score** | **2023** | **2022** | **With zip bag** | **1** | **42** | **14** | **4.74** | **HT1_LG14_27543259** | **49.2** | **17.2** |
| **Phenotyping score** | **2023** | **2022** | **With zip bag** | **2** | **14** | **1** | **7.66** | **HT1_LG01_24114628** | **16.9** | **37.7** |
| Phenotyping score | 2023 | 2022 | With zip bag | 2 | 21 | 1 | 4.89 | HT1_LG01_24856212 | 19.5 | 20.9 |
| **Phenotyping score** | **2023** | **2022** | **With zip bag** | **3** | **21** | **1** | **4.38** | **HT1_LG01_29924205** | **43** | **73.5** |
| **Phenotyping score** | **2023** | **2022** | **With zip bag** | **1-3** | **14** | **1** | **7.67** | **HT1_LG01_24396591** | **17.8** | **38.7** |
| Phenotyping score | 2023 | 2022 | With zip bag | 1-3 | 21 | 1 | 6.22 | HT1_LG01_24856212 | 19.5 | 27.9 |
| Phenotyping score | 2023 | 2022 | With zip bag | 1-3 | 28 | 1 | 4.53 | HT1_LG01_29002287 | 40.5 | 21.8 |
| **Phenotyping score** | **2023** | **2022** | **With zip bag** | **1-3** | **28** | **3** | **4.50** | **HT1_LG03_37007502** | **78.6** | **19.0** |
| **Phenotyping score** | **2023** | **2022** | **With zip bag** | **1-3** | **35** | **14** | **4.95** | **HT1_LG14_27543259** | **49.2** | **17.6** |
| Phenotyping score | 2023 | 2022 | With zip bag | 1-3 | 42 | 14 | 4.80 | HT1_LG14_27543259 | 49.2 | 17.1 |
| **Phenotyping score** | **2023** | **2022** | **With zip bag** | **1-3** | **70** | **4** | **5.04** | **Hi07b02** | **30.0** | **18.9** |
| **Phenotyping score** | **2023** | **2022** | **Without zip bag** | **1** | **21** | **14** | **4.53** | **HT1_LG14_19111015** | **19.3** | **17.5** |
| Phenotyping score | 2023 | 2022 | Without zip bag | 2 | 35 | 2 | 5.18 | HT1_LG02_8740212 | 30.6 | 20.4 |
| **Phenotyping score** | **2023** | **2022** | **Without zip bag** | **2** | **42** | **2** | **5.39** | **HT1_LG02_8740212** | **30.6** | **21.4** |
| **Phenotyping score** | **2023** | **2022** | **Without zip bag** | **3** | **28** | **2** | **4.47** | **HT1_LG02_16066858** | **43.0** | **20.4** |
| Phenotyping score | 2023 | 2022 | Without zip bag | 1-3 | 21 | 14 | 4.42 | HT1_LG14_28024105 | 52.3 | 15.9 |
| Phenotyping score | 2023 | 2022 | Without zip bag | 1-3 | 28 | 14 | 4.54 | HT1_LG14_29010646 | 55.8 | 17.7 |
| Phenotyping score | 2023 | 2022 | Without zip bag | 1-3 | 35 | 1 | 4.29 | HT1_LG01_25603900 | 22.2 | 17.0 |
| **Phenotyping score** | **2023** | **2022** | **Without zip bag** | **1-3** | **35** | **14** | **5.09** | **HT1_LG14_28024105 (4.93)** | **52.3** | **19.8** |
| **Phenotyping score** | **2023** | **2022** | **Without zip bag** | **1-3** | **42** | **1** | **4.38** | **HT1_LG01_25603900** | **22.2** | **17.9** |
| **Phenotyping score** | **2025** | **2022** | **With zip bag** | **4** | **77** | **6** | **4.33** | **HT1_LG06_11098342 (4.23)** | **14.3** | **16.4** |
| Phenotyping score | 2025 | 2022, 2023 | With zip bag | 4-5 | 14 | 4 | 4.63 | HT1_LG04_7914653 | 13.6 | 18.0 |
| **Phenotyping score** | **2025** | **2022, 2023** | **With zip bag** | **4-5** | **35** | **4** | **4.73** | **HT1_LG04_23637976** | **27.1** | **18.3** |
| **Phenotyping score** | **2025** | **2022, 2023** | **With zip bag** | **4-5** | **35** | **13** | **4.65** | **HT1_LG13_14598871** | **60.3** | **19.7** |
| **Phenotyping score** | **2023, 2025** | **2022** | **With zip bag** | **1-4** | **14** | **1** | **6.89** | **HT1_LG01_24396591** | **17.8** | **32.0** |
| Phenotyping score | 2023, 2025 | 2022 | With zip bag | 1-4 | 21 | 1 | 4.94 | HT1_LG01_25603900 (4.93) | 20.5 | 21.5 |
| **Phenotyping score** | **2023, 2025** | **2022** | **With zip bag** | **1-4** | **28** | **13** | **4.33** | **HT1_LG13_14598871** | **60.3** | **16.7** |
| Phenotyping score | 2023, 2025 | 2022 | With zip bag | 1-4 | 35 | 13 | 4.20 | HT1_LG13_13516207 | 58.7 | 16.2 |
| **Phenotyping score** | **2023, 2025** | **2022, 2023** | **With zip bag** | **1-5** | **14** | **1** | **7.95** | **HT1_LG01_24856212** | **19.5** | **30.8** |
| Phenotyping score | 2023, 2025 | 2022, 2023 | With zip bag | 1-5 | 21 | 1 | 6.14 | HT1_LG01_25603900 (6.12) | 21.5 | 24.0 |
| **Phenotyping score** | **2023, 2025** | **2022, 2023** | **With zip bag** | **1-5** | **28** | **2** | **5.61** | **HT1_LG02_8740212** | **30.6** | **21.8** |
| **Phenotyping score** | **2023, 2025** | **2022, 2023** | **With zip bag** | **1-5** | **28** | **13** | **4.94** | **HT1_LG13_13516207 (4.93)** | **58.3** | **19.5** |
| Phenotyping score | 2023, 2025 | 2022, 2023 | With zip bag | 1-5 | 35 | 2 | 4.50 | HT1_LG02_8740212 | 30.6 | 17.7 |
| Phenotyping score | 2023, 2025 | 2022, 2023 | With zip bag | 1-5 | 35 | 13 | 4.36 | HT1_LG13_14253854 | 59.4 | 18.0 |
| Phenotyping score | 2023, 2025 | 2022, 2023 | With zip bag | 1-5 | 77 | 12 | 4.52 | HT1_LG12_21486850 | 27.5 | 16.2 |
| **Phenotyping score** | **2023, 2025** | **2022, 2023** | **With zip bag** | **1-5** | **84** | **12** | **4.86** | **HT1_LG12_21486850** | **27.5** | **17.3** |
| AUDPC of phenotyping score | 2023 | 2022 | With zip bag | 1 | 28 | 14 | 4.26 | HT1_LG14_19111015 | 19.3 | 16.2 |
| AUDPC of phenotyping score | 2023 | 2022 | With zip bag | 1 | 35 | 14 | 4.55 | HT1_LG14_19111015 | 19.3 | 17.2 |
| AUDPC of phenotyping score | 2023 | 2022 | With zip bag | 1 | 42 | 14 | 4.41 | HT1_LG14_19111015 | 19.3 | 16.7 |
| AUDPC of phenotyping score | 2023 | 2022 | With zip bag | 1 | 49 | 14 | 4.52 | HT1_LG14_27543259 | 49.2 | 16.4 |
| AUDPC of phenotyping score | 2023 | 2022 | With zip bag | 1 | 56 | 14 | 4.65 | HT1_LG14_19111015 | 19.3 | 17.6 |
| **AUDPC of phenotyping score** | **2023** | **2022** | **With zip bag** | **1** | **63** | **14** | **4.85** | **HT1_LG14_19111015** | **19.3** | **18.3** |
| AUDPC of phenotyping score | 2023 | 2022 | With zip bag | 1 | 70 | 14 | 4.84 | HT1_LG14_19111015 | 19.3 | 18.3 |
| AUDPC of phenotyping score | 2023 | 2022 | With zip bag | 1 | 77 | 14 | 4.69 | HT1_LG14_19111015 | 19.3 | 17.8 |
| AUDPC of phenotyping score | 2023 | 2022 | With zip bag | 1 | 84 | 14 | 4.5 | HT1_LG14_19111015 | 19.3 | 17.1 |
| AUDPC of phenotyping score | 2023 | 2022 | With zip bag | 1 | 91 | 14 | 4.32 | HT1_LG14_19111015 | 19.3 | 16.5 |
| AUDPC of phenotyping score | 2023 | 2022 | With zip bag | 2 | 14 | 1 | 7.66 | HT1_LG01_24114628 | 16.9 | 37.7 |
| **AUDPC of phenotyping score** | **2023** | **2022** | **With zip bag** | **2** | **21** | **1** | **8.37** | **HT1_LG01_24396591** | **17.8** | **39.1** |
| AUDPC of phenotyping score | 2023 | 2022 | With zip bag | 2 | 28 | 1 | 7.89 | HT1_LG01_24396591 | 17.8 | 37.1 |
| AUDPC of phenotyping score | 2023 | 2022 | With zip bag | 2 | 35 | 1 | 6.96 | HT1_LG01_24396591 | 17.8 | 32.7 |
| AUDPC of phenotyping score | 2023 | 2022 | With zip bag | 2 | 42 | 1 | 6.11 | HT1_LG01_24396591 | 17.8 | 28.3 |
| AUDPC of phenotyping score | 2023 | 2022 | With zip bag | 2 | 49 | 1 | 5.69 | HT1_LG01_25603900 | 22.2 | 22.7 |
| AUDPC of phenotyping score | 2023 | 2022 | With zip bag | 2 | 49 | 14 | 4.21 | HT1_LG14_28024105 | 52.3 | 15.5 |
| AUDPC of phenotyping score | 2023 | 2022 | With zip bag | 2 | 56 | 1 | 5.50 | HT1_LG01_25603900 | 22.2 | 21.6 |
| AUDPC of phenotyping score | 2023 | 2022 | With zip bag | 2 | 63 | 1 | 5.31 | HT1_LG01_25603900 | 22.2 | 20.8 |
| AUDPC of phenotyping score | 2023 | 2022 | With zip bag | 2 | 63 | 14 | 4.27 | HT1_LG14_27543259 | 49.2 | 15.7 |
| AUDPC of phenotyping score | 2023 | 2022 | With zip bag | 2 | 70 | 1 | 5.13 | HT1_LG01_25603900 | 22.2 | 20.1 |
| AUDPC of phenotyping score | 2023 | 2022 | With zip bag | 2 | 77 | 1 | 5.00 | HT1_LG01_25603900 | 22.2 | 19.5 |
| AUDPC of phenotyping score | 2023 | 2022 | With zip bag | 2 | 84 | 1 | 4.92 | HT1_LG01_25603900 | 22.2 | 19.2 |
| AUDPC of phenotyping score | 2023 | 2022 | With zip bag | 2 | 91 | 1 | 4.86 | HT1_LG01_25603900 | 22.2 | 19.0 |
| **AUDPC of phenotyping score** | **2023** | **2022** | **With zip bag** | **2** | **91** | **14** | **4.30** | **HT1_LG14_27543259** | **49.2** | **15.8** |
| AUDPC of phenotyping score | 2023 | 2022 | With zip bag | 1-3 | 14 | 1 | 7.67 | HT1_LG01_24396591 | 17.8 | 38.7 |
| **AUDPC of phenotyping score** | **2023** | **2022** | **With zip bag** | **1-3** | **21** | **1** | **8.25** | **HT1_LG01_24396591** | **17.8** | **40.7** |
| AUDPC of phenotyping score | 2023 | 2022 | With zip bag | 1-3 | 28 | 1 | 8.16 | HT1_LG01_24396591 | 17.8 | 40.3 |
| AUDPC of phenotyping score | 2023 | 2022 | With zip bag | 1-3 | 35 | 1 | 7.76 | HT1_LG01_24396591 | 17.8 | 38.5 |
| AUDPC of phenotyping score | 2023 | 2022 | With zip bag | 1-3 | 35 | 14 | 4.38 | HT1_LG14_28024105 | 52.3 | 15.7 |
| AUDPC of phenotyping score | 2023 | 2022 | With zip bag | 1-3 | 35 | 14 | 4.31 | HT1_LG14_27543259 | 49.2 | 15.5 |
| AUDPC of phenotyping score | 2023 | 2022 | With zip bag | 1-3 | 42 | 1 | 7.28 | HT1_LG01_24396591 | 17.8 | 36.1 |
| AUDPC of phenotyping score | 2023 | 2022 | With zip bag | 1-3 | 42 | 14 | 4.83 | HT1_LG14_27543259 | 49.2 | 17.2 |
| AUDPC of phenotyping score | 2023 | 2022 | With zip bag | 1-3 | 49 | 1 | 6.91 | HT1_LG01_25603900 (6.90) | 21.5 | 30.06 |
| AUDPC of phenotyping score | 2023 | 2022 | With zip bag | 1-3 | 49 | 14 | 5.15 | HT1_LG14_27543259 | 49.2 | 18.2 |
| AUDPC of phenotyping score | 2023 | 2022 | With zip bag | 1-3 | 56 | 1 | 6.72 | HT1_LG01_25603900 | 22.2 | 27.8 |
| AUDPC of phenotyping score | 2023 | 2022 | With zip bag | 1-3 | 56 | 14 | 5.37 | HT1_LG14_27543259 | 49.2 | 18.9 |
| AUDPC of phenotyping score | 2023 | 2022 | With zip bag | 1-3 | 63 | 1 | 6.55 | HT1_LG01_25603900 | 22.2 | 27.0 |
| AUDPC of phenotyping score | 2023 | 2022 | With zip bag | 1-3 | 63 | 14 | 5.52 | HT1_LG14_27543259 | 49.2 | 19.4 |
| AUDPC of phenotyping score | 2023 | 2022 | With zip bag | 1-3 | 70 | 1 | 6.38 | HT1_LG01_25603900 | 22.2 | 26.1 |
| AUDPC of phenotyping score | 2023 | 2022 | With zip bag | 1-3 | 70 | 14 | 5.60 | HT1_LG14_27543259 | 49.2 | 19.6 |
| AUDPC of phenotyping score | 2023 | 2022 | With zip bag | 1-3 | 77 | 1 | 6.24 | HT1_LG01_25603900 | 22.2 | 25.5 |
| AUDPC of phenotyping score | 2023 | 2022 | With zip bag | 1-3 | 77 | 14 | 5.65 | HT1_LG14_27543259 | 49.2 | 19.8 |
| AUDPC of phenotyping score | 2023 | 2022 | With zip bag | 1-3 | 84 | 1 | 6.12 | HT1_LG01_25603900 | 22.2 | 24.9 |
| **AUDPC of phenotyping score** | **2023** | **2022** | **With zip bag** | **1-3** | **84** | **14** | **5.66** | **HT1_LG14_27543259** | **49.2** | **19.8** |
| AUDPC of phenotyping score | 2023 | 2022 | With zip bag | 1-3 | 91 | 1 | 6.00 | HT1_LG01_25603900 | 22.2 | 24.4 |
| AUDPC of phenotyping score | 2023 | 2022 | With zip bag | 1-3 | 91 | 14 | 5.63 | HT1_LG14_27543259 | 49.2 | 19.7 |
| AUDPC of phenotyping score | 2023 | 2022 | Without zip bag | 1 | 49 | 14 | 4.33 | HT1_LG14_27543259 | 49.2 | 15.5 |
| AUDPC of phenotyping score | 2023 | 2022 | Without zip bag | 1 | 56 | 14 | 4.49 | HT1_LG14_27543259 | 49.2 | 16.1 |
| **AUDPC of phenotyping score** | **2023** | **2022** | **Without zip bag** | **1** | **63** | **14** | **4.57** | **HT1_LG14_27543259** | **49.2** | **16.3** |
| AUDPC of phenotyping score | 2023 | 2022 | Without zip bag | 1 | 70 | 14 | 4.54 | HT1_LG14_27543259 | 49.2 | 16.2 |
| AUDPC of phenotyping score | 2023 | 2022 | Without zip bag | 1 | 77 | 14 | 4.44 | HT1_LG14_27543259 | 49.2 | 15.9 |
| AUDPC of phenotyping score | 2023 | 2022 | Without zip bag | 1 | 84 | 14 | 4.36 | HT1_LG14_27543259 | 49.2 | 15.7 |
| AUDPC of phenotyping score | 2023 | 2022 | Without zip bag | 1 | 91 | 14 | 4.35 | HT1_LG14_27543259 | 49.2 | 15.6 |
| AUDPC of phenotyping score | 2023 | 2022 | Without zip bag | 2 | 42 | 2 | 4.83 | HT1_LG02_8740212 (4.73) | 30.4 | 18.7 |
| **AUDPC of phenotyping score** | **2023** | **2022** | **Without zip bag** | **2** | **49** | **2** | **5.15** | **HT1_LG02_8740212 (5.10)** | **30.4** | **19.8** |
| AUDPC of phenotyping score | 2023 | 2022 | Without zip bag | 2 | 56 | 2 | 5.02 | HT1_LG02_8740212 | 30.6 | 19.5 |
| AUDPC of phenotyping score | 2023 | 2022 | Without zip bag | 2 | 63 | 2 | 4.91 | HT1_LG02_8740212 | 30.6 | 19.0 |
| AUDPC of phenotyping score | 2023 | 2022 | Without zip bag | 2 | 70 | 2 | 4.77 | HT1_LG02_8740212 | 30.6 | 18.5 |
| AUDPC of phenotyping score | 2023 | 2022 | Without zip bag | 2 | 77 | 2 | 4.57 | HT1_LG02_8740212 | 30.6 | 17.7 |
| AUDPC of phenotyping score | 2023 | 2022 | Without zip bag | 2 | 84 | 2 | 4.38 | HT1_LG02_8740212 | 30.6 | 17.0 |
| AUDPC of phenotyping score | 2023 | 2022 | Without zip bag | 2 | 91 | 2 | 4.21 | HT1_LG02_8893221 | 30.6 | 16.4 |
| AUDPC of phenotyping score | 2023 | 2022 | Without zip bag | 1-3 | 42 | 1 | 4.48 | HT1_LG01_24856212 | 19.5 | 19.9 |
| AUDPC of phenotyping score | 2023 | 2022 | Without zip bag | 1-3 | 42 | 14 | 4.80 | HT1_LG14_28024105 | 52.3 | 17.1 |
| **AUDPC of phenotyping score** | **2023** | **2022** | **Without zip bag** | **1-3** | **49** | **1** | **4.57** | **HT1_LG01_24856212** | **19.5** | **20.1** |
| AUDPC of phenotyping score | 2023 | 2022 | Without zip bag | 1-3 | 49 | 14 | 4.97 | HT1_LG14_28024105 | 52.3 | 17.6 |
| AUDPC of phenotyping score | 2023 | 2022 | Without zip bag | 1-3 | 56 | 1 | 4.52 | HT1_LG01_24856212 | 19.5 | 19.8 |
| AUDPC of phenotyping score | 2023 | 2022 | Without zip bag | 1-3 | 56 | 14 | 5.06 | HT1_LG14_28024105 | 52.3 | 17.9 |
| AUDPC of phenotyping score | 2023 | 2022 | Without zip bag | 1-3 | 63 | 1 | 4.46 | HT1_LG01_24856212 | 19.5 | 19.5 |
| **AUDPC of phenotyping score** | **2023** | **2022** | **Without zip bag** | **1-3** | **63** | **14** | **5.09** | **HT1_LG14_28024105** | **52.3** | **18.0** |
| AUDPC of phenotyping score | 2023 | 2022 | Without zip bag | 1-3 | 70 | 1 | 4.41 | HT1_LG01_24856212 | 19.5 | 19.2 |
| AUDPC of phenotyping score | 2023 | 2022 | Without zip bag | 1-3 | 70 | 14 | 5.06 | HT1_LG14_28024105 | 52.3 | 17.9 |
| AUDPC of phenotyping score | 2023 | 2022 | Without zip bag | 1-3 | 77 | 14 | 5.02 | HT1_LG14_28024105 | 52.3 | 17.8 |
| AUDPC of phenotyping score | 2023 | 2022 | Without zip bag | 1-3 | 84 | 1 | 4.34 | HT1_LG01_24856212 | 19.5 | 18.7 |
| AUDPC of phenotyping score | 2023 | 2022 | Without zip bag | 1-3 | 84 | 14 | 4.97 | HT1_LG14_28024105 | 52.3 | 17.6 |
| AUDPC of phenotyping score | 2023 | 2022 | Without zip bag | 1-3 | 91 | 1 | 4.30 | HT1_LG01_24856212 | 19.5 | 18.4 |
| AUDPC of phenotyping score | 2023 | 2022 | Without zip bag | 1-3 | 91 | 14 | 4.90 | HT1_LG14_28024105 | 52.3 | 17.4 |
| **AUDPC of phenotyping score** | **2025** | **2022, 2023** | **With zip bag** | **4-5** | **14** | **4** | **4.63** | **HT1_LG04_7914653** | **13.6** | **18.0** |
| AUDPC of phenotyping score | 2023, 2025 | 2022 | With zip bag | 1-4 | 14 | 1 | 6.89 | HT1_LG01_24396591 | 17.8 | 32.0 |
| **AUDPC of phenotyping score** | **2023, 2025** | **2022** | **With zip bag** | **1-4** | **21** | **1** | **6.98** | **HT1_LG01_24856212** | **19.5** | **30.0** |
| AUDPC of phenotyping score | 2023, 2025 | 2022 | With zip bag | 1-4 | 28 | 1 | 6.51 | HT1_LG01_24856212 | 19.5 | 28.0 |
| AUDPC of phenotyping score | 2023, 2025 | 2022 | With zip bag | 1-4 | 35 | 1 | 5.85 | HT1_LG01_24856212 (5.82) | 20.5 | 25.1 |
| AUDPC of phenotyping score | 2023, 2025 | 2022 | With zip bag | 1-4 | 42 | 1 | 5.19 | HT1_LG01_25603900 (5.18) | 21.5 | 21.9 |
| AUDPC of phenotyping score | 2023, 2025 | 2022 | With zip bag | 1-4 | 42 | 13 | 4.32 | HT1_LG13_13516207 | 58.7 | 16.7 |
| AUDPC of phenotyping score | 2023, 2025 | 2022 | With zip bag | 1-4 | 42 | 14 | 4.2 | HT1_LG14_27543259 | 49.2 | 15.1 |
| AUDPC of phenotyping score | 2023, 2025 | 2022 | With zip bag | 1-4 | 49 | 1 | 4.53 | HT1_LG01_25603900 (4.53) | 21.5 | 19.3 |
| AUDPC of phenotyping score | 2023, 2025 | 2022 | With zip bag | 1-4 | 49 | 13 | 4.45 | HT1_LG13_13516207 | 58.7 | 17.2 |
| AUDPC of phenotyping score | 2023, 2025 | 2022 | With zip bag | 1-4 | 49 | 14 | 4.38 | HT1_LG14_27543259 | 49.2 | 15.7 |
| **AUDPC of phenotyping score** | **2023, 2025** | **2022** | **With zip bag** | **1-4** | **56** | **13** | **4.46** | **HT1_LG13_13516207** | **58.7** | **17.2** |
| **AUDPC of phenotyping score** | **2023, 2025** | **2022** | **With zip bag** | **1-4** | **56** | **14** | **4.42** | **HT1_LG14_27543259** | **49.2** | **15.8** |
| **AUDPC of phenotyping score** | **2023, 2025** | **2022** | **With zip bag** | **1-4** | **63** | **13** | **4.46** | **HT1_LG13_13516207** | **58.7** | **17.1** |
| AUDPC of phenotyping score | 2023, 2025 | 2022 | With zip bag | 1-4 | 63 | 14 | 4.33 | HT1_LG14_27543259 | 49.2 | 15.6 |
| AUDPC of phenotyping score | 2023, 2025 | 2022 | With zip bag | 1-4 | 70 | 13 | 4.39 | HT1_LG13_13516207 | 58.7 | 16.9 |
| AUDPC of phenotyping score | 2023, 2025 | 2022 | With zip bag | 1-4 | 77 | 13 | 4.20 | HT1_LG13_13516207 | 58.7 | 16.2 |
| AUDPC of phenotyping score | 2023, 2025 | 2022, 2023 | With zip bag | 1-5 | 14 | 1 | 7.95 | HT1_LG01_24856212 | 19.5 | 30.8 |
| **AUDPC of phenotyping score** | **2023, 2025** | **2022, 2023** | **With zip bag** | **1-5** | **21** | **1** | **8.37** | **HT1_LG01_24856212** | **19.5** | **32.4** |
| AUDPC of phenotyping score | 2023, 2025 | 2022, 2023 | With zip bag | 1-5 | 28 | 1 | 7.80 | HT1_LG01_24856212 (7.77) | 20.5 | 30.4 |
| AUDPC of phenotyping score | 2023, 2025 | 2022, 2023 | With zip bag | 1-5 | 35 | 1 | 6.80 | HT1_LG01_25603900 (6.76) | 21.5 | 26.2 |
| AUDPC of phenotyping score | 2023, 2025 | 2022, 2023 | With zip bag | 1-5 | 42 | 1 | 5.97 | HT1_LG01_25603900 (5.96) | 21.5 | 23.2 |
| AUDPC of phenotyping score | 2023, 2025 | 2022, 2023 | With zip bag | 1-5 | 49 | 1 | 5.38 | HT1_LG01_25603900 | 22.2 | 20.6 |
| AUDPC of phenotyping score | 2023, 2025 | 2022, 2023 | With zip bag | 1-5 | 49 | 2 | 4.32 | HT1_LG02_8740212 | 30.6 | 16.6 |
| AUDPC of phenotyping score | 2023, 2025 | 2022, 2023 | With zip bag | 1-5 | 56 | 1 | 4.86 | HT1_LG01_25603900 | 22.2 | 18.8 |
| **AUDPC of phenotyping score** | **2023, 2025** | **2022, 2023** | **With zip bag** | **1-5** | **56** | **2** | **4.40** | **HT1_LG02_8740212** | **30.6** | **17.0** |
| AUDPC of phenotyping score | 2023, 2025 | 2022, 2023 | With zip bag | 1-5 | 63 | 1 | 4.44 | HT1_LG01_25603900 | 22.2 | 17.3 |
| AUDPC of phenotyping score | 2023, 2025 | 2022, 2023 | With zip bag | 1-5 | 63 | 2 | 4.39 | HT1_LG02_8740212 | 30.6 | 17.1 |
| AUDPC of phenotyping score | 2023, 2025 | 2022, 2023 | With zip bag | 1-5 | 70 | 1 | 4.13 | HT1_LG01_25603900 | 22.2 | 16.2 |
| AUDPC of phenotyping score | 2023, 2025 | 2022, 2023 | With zip bag | 1-5 | 70 | 2 | 4.27 | HT1_LG02_8740212 | 30.6 | 16.8 |
| **AUDPC of phenotyping score** | **2023, 2025** | **2022, 2023** | **With zip bag** | **1-5** | **70** | **13** | **4.11** | **HT1_LG13_14253854** | **59.4** | **16.7** |

For each unique combination, the row corresponding to the marker with the highest logarithm of the odds (LOD) score per linkage group (LG) across all time points is highlighted in bold. * LOD score of the respective marker is given in parentheses if a higher interpolated LOD score exists. AUDPC: area under the disease progress curve; cM: centimorgan; dpi: days post-inoculation.

**Table S4.** Cullis broad-sense heritability estimates for the F_1_ population across all repetitions of the detached leaf assay

| **Trait** | **Leaf origin** | **dpi** | **Cullis broad-sense heritability** | **Genetic variance** | **Residual variance** |
| --- | --- | --- | --- | --- | --- |
| Necrotic spots per dm^2^ | Field | 7 | 0.1504041 | 41.75649 | 928.4264 |
| Necrotic spots per dm^2^ | Field | 10 | 0.1191345 | 238.1744 | 6934.096 |
| Necrotic spots per dm^2^ | Field | 14 | 0.1198467 | 243.0972 | 7029.588 |
| Necrotic spots per dm^2^ | Greenhouse | 7 | 0.4336041 | 50.48661 | 258.1869 |
| Necrotic spots per dm^2^ | Greenhouse | 10 | 0.4460849 | 402.5932 | 1956.298 |
| Necrotic spots per dm^2^ | Greenhouse | 14 | 0.4638069 | 950.0199 | 4296.528 |
| AUDPC of necrotic spots per dm^2^ | Field | 7 | 0.1504041 | 511.517 | 11373.22 |
| AUDPC of necrotic spots per dm^2^ | Field | 10 | 0.1498459 | 2762.225 | 61683.53 |
| AUDPC of necrotic spots per dm^2^ | Field | 14 | 0.1335724 | 11199.76 | 286001.7 |
| AUDPC of necrotic spots per dm^2^ | Greenhouse | 7 | 0.4336041 | 618.461 | 3162.789 |
| AUDPC of necrotic spots per dm^2^ | Greenhouse | 10 | 0.4449213 | 3715.189 | 18138.15 |
| AUDPC of necrotic spots per dm^2^ | Greenhouse | 14 | 0.4507839 | 23598.64 | 112497.7 |
| Percentage of necrotic leaf area | Field | 7 | 0.6771201 | 0.2453238 | 0.4526842 |
| Percentage of necrotic leaf area | Field | 10 | 0.2875233 | 0.2150126 | 2.092825 |
| Percentage of necrotic leaf area | Field | 14 | 0.1353804 | 2.272969 | 57.16085 |
| Percentage of necrotic leaf area | Greenhouse | 7 | 0.6585808 | 0.9076465 | 1.822341 |
| Percentage of necrotic leaf area | Greenhouse | 10 | 0.662712 | 1.029062 | 2.027813 |
| Percentage of necrotic leaf area | Greenhouse | 14 | 0.5385138 | 1.62573 | 5.433614 |
| AUDPC of percentage necrotic leaf area | Field | 7 | 0.6771201 | 3.005216 | 5.545382 |
| AUDPC of percentage necrotic leaf area | Field | 10 | 0.62585 | 10.16564 | 23.60406 |
| AUDPC of percentage necrotic leaf area | Field | 14 | 0.2035001 | 26.69398 | 411.2589 |
| AUDPC of percentage necrotic leaf area | Greenhouse | 7 | 0.6585808 | 11.11867 | 22.32367 |
| AUDPC of percentage necrotic leaf area | Greenhouse | 10 | 0.6602171 | 39.4135 | 78.54931 |
| AUDPC of percentage necrotic leaf area | Greenhouse | 14 | 0.6480424 | 114.7325 | 241.525 |

AUDPC: area under the disease progress curve; dpi: days post-inoculation.

**Table S5.** Cullis broad-sense heritability estimates for the F_1_ population across all repetitions of the greenhouse experiment

| **Trait** | **dpi** | **Cullis broad-sense heritability** | **Genetic variance** | **Residual variance** |
| --- | --- | --- | --- | --- |
| Phenotyping score | 14 | 0.374682 | 0.072363986 | 0.5674629 |
| Phenotyping score | 21 | 0.5811445 | 0.18111184 | 0.6071584 |
| Phenotyping score | 28 | 0.4847523 | 0.09942568 | 0.4942194 |
| Phenotyping score | 35 | 0.4702627 | 0.08540369 | 0.450214 |
| Phenotyping score | 42 | 0.4236039 | 0.06060667 | 0.3867077 |
| Phenotyping score | 49 | 0.3222033 | 0.03526517 | 0.3491716 |
| Phenotyping score | 56 | 0.3434378 | 0.0311252 | 0.2798583 |
| Phenotyping score | 63 | 0.320749 | 0.02522438 | 0.2514346 |
| Phenotyping score | 70 | 0.3158693 | 0.02263535 | 0.2307946 |
| Phenotyping score | 77 | 0.2819356 | 0.01813437 | 0.2176707 |
| Phenotyping score | 84 | 0.2639052 | 0.01579507 | 0.2077453 |
| Phenotyping score | 91 | 0.1751597 | 0.00788327 | 0.1754817 |
| AUDPC of phenotyping score | 14 | 0.374682 | 3.545835 | 27.80568 |
| AUDPC of phenotyping score | 21 | 0.4977997 | 19.440615 | 91.66156 |
| AUDPC of phenotyping score | 28 | 0.5713502 | 49.320402 | 172.20314 |
| AUDPC of phenotyping score | 35 | 0.590868 | 82.783943 | 266.40992 |
| AUDPC of phenotyping score | 42 | 0.5951207 | 119.077998 | 376.39379 |
| AUDPC of phenotyping score | 49 | 0.5865858 | 151.852811 | 497.52425 |
| AUDPC of phenotyping score | 56 | 0.5740541 | 179.946997 | 621.20886 |
| AUDPC of phenotyping score | 63 | 0.5734046 | 210.772316 | 729.57462 |
| AUDPC of phenotyping score | 70 | 0.5797834 | 245.624423 | 827.92343 |
| AUDPC of phenotyping score | 77 | 0.5852426 | 281.084204 | 926.06177 |
| AUDPC of phenotyping score | 84 | 0.5903743 | 317.043305 | 1022.26974 |
| AUDPC of phenotyping score | 91 | 0.5934786 | 350.406133 | 1115.16393 |

AUDPC: area under the disease progress curve; dpi: days post-inoculation.

# Supplementary Scripts

**Supplementary Script S1.** Leaf_area_calculator.py

""" @author: Matthias Pfeifer """

import cv2

import numpy as np

import os

import glob

import pandas as pd

from concurrent.futures import ThreadPoolExecutor

# Function to resize the image

def resize_image(image, scale_percent=40):

width = int(image.shape[1] * scale_percent / 100)

height = int(image.shape[0] * scale_percent / 100)

return cv2.resize(image, (width, height), interpolation=cv2.INTER_AREA)

# Function to extract the leaf from the image

def extract_leaf(image):

lower_green = np.array([30, 50, 50])

upper_green = np.array([90, 255, 255])

lower_brown = np.array([0, 20, 7])

upper_brown = np.array([40, 94, 30])

lower_leaf = np.array([min(lower_green[0], lower_brown[0]),

min(lower_green[1], lower_brown[1]),

min(lower_green[2], lower_brown[2])])

upper_leaf = np.array([max(upper_green[0], upper_brown[0]),

max(upper_green[1], upper_brown[1]),

max(upper_green[2], upper_brown[2])])

hsv = cv2.cvtColor(image, cv2.COLOR_BGR2HSV)

mask = cv2.inRange(hsv, lower_leaf, upper_leaf)

contours, _ = cv2.findContours(mask, cv2.RETR_EXTERNAL, cv2.CHAIN_APPROX_SIMPLE)

if contours:

max_contour = max(contours, key=cv2.contourArea)

cv2.drawContours(image, [max_contour], -1, (0, 255, 0), thickness=10)

return image, max_contour

else:

print("No contours found for the image.")

return None, None

# Function to find a rectangular structure in the image (adjusted for light blue)

def find_rectangular_structure(image):

# Convert to HSV

hsv = cv2.cvtColor(image, cv2.COLOR_BGR2HSV)

# Define the color range for light blue (adjust if needed)

lower_blue = np.array([80, 50, 50])

upper_blue = np.array([130, 255, 255])

# Create a mask for the blue color

mask = cv2.inRange(hsv, lower_blue, upper_blue)

# Find contours in the masked image

contours, _ = cv2.findContours(mask, cv2.RETR_EXTERNAL, cv2.CHAIN_APPROX_SIMPLE)

tolerance = 0.1 # 10% Tolerance for approximation

best_rect = None

best_area = 0

for contour in contours:

epsilon = tolerance * cv2.arcLength(contour, True)

approx = cv2.approxPolyDP(contour, epsilon, True)

if len(approx) == 4:

x, y, w, h = cv2.boundingRect(approx)

aspect_ratio = w / float(h)

# Check if the contour is roughly rectangular

if 0.9 <= aspect_ratio <= 1.1:

area = w * h

total_area = image.shape[0] * image.shape[1]

# Ensure the rectangle is neither too small nor too large

if 0.01 * total_area <= area <= 0.30 * total_area and area > best_area:

best_area = area

best_rect = (x, y, w, h)

if best_rect is not None:

x, y, w, h = best_rect

cv2.rectangle(image, (x, y), (x + w, y + h), (0, 0, 255), 10)

return image, best_rect

else:

print("No rectangular structure found.")

return None, None

input_folder = 'input_images'

output_folder = 'output_images_leafarea'

def process_image(image_path):

image = cv2.imread(image_path)

image = resize_image(image, scale_percent=40)

piece_image, piece_bbox = find_rectangular_structure(image)

leaf_image, leaf_contour = extract_leaf(image)

filename = os.path.basename(image_path)

output_filename = os.path.splitext(filename)[0] + '_piece.jpg'

output_path = os.path.join(output_folder, output_filename)

cv2.imwrite(output_path, image, [int(cv2.IMWRITE_JPEG_QUALITY), 40]) # Set JPG quality to 40

print(output_filename + " saved.")

return filename, leaf_contour, piece_bbox

os.makedirs(output_folder, exist_ok=True)

# Limit the number of threads to 10

max_threads = 10

with ThreadPoolExecutor(max_workers=max_threads) as executor:

processed_images = list(executor.map(process_image, glob.glob(os.path.join(input_folder, '*.jpg'))))

print("Process completed.")

data = {'Image_name': [], 'Piecearea': [], 'Leafarea': [], 'Leafarea_calculated': []}

for filename, leaf_contour, piece_bbox in processed_images:

if leaf_contour is not None and piece_bbox is not None:

data['Image_name'].append(filename)

leaf_area = cv2.contourArea(leaf_contour)

x, y, w, h = piece_bbox

piece_area = w * h

data['Piecearea'].append(piece_area)

data['Leafarea'].append(leaf_area)

data['Leafarea_calculated'].append((leaf_area / piece_area) * 4.00)

else:

data['Image_name'].append(filename)

data['Piecearea'].append("NA")

data['Leafarea'].append("NA")

data['Leafarea_calculated'].append("NA")

df = pd.DataFrame(data)

excel_file = 'Output-file.xlsx'

df.to_excel(excel_file, index=False)

print("Excel file created", excel_file

**Supplementary Script 2.** Leaf_extractor_resizer.py

""" @author: Matthias Pfeifer """

import cv2

import numpy as np

import os

import glob

import openpyxl

from concurrent.futures import ThreadPoolExecutor

def extract_leaf(image):

# Define the extended range for the entire leaf in the HSV color space

lower_green = np.array([30, 50, 50])

upper_green = np.array([90, 255, 255])

lower_brown = np.array([0, 20, 7])

upper_brown = np.array([40, 94, 30])

lower_leaf = np.array([min(lower_green[0], lower_brown[0]),

min(lower_green[1], lower_brown[1]),

min(lower_green[2], lower_brown[2])])

upper_leaf = np.array([max(upper_green[0], upper_brown[0]),

max(upper_green[1], upper_brown[1]),

max(upper_green[2], upper_brown[2])])

hsv = cv2.cvtColor(image, cv2.COLOR_BGR2HSV)

mask = cv2.inRange(hsv, lower_leaf, upper_leaf)

# Extracts the largest object/leaf by selecting the largest contour based on area.

contours, _ = cv2.findContours(mask, cv2.RETR_EXTERNAL, cv2.CHAIN_APPROX_SIMPLE)

if contours:

max_contour = max(contours, key=cv2.contourArea)

mask = np.zeros(image.shape[:2], dtype=np.uint8)

cv2.drawContours(mask, [max_contour], -1, (255), thickness=cv2.FILLED)

leaf_with_transparent_bg = cv2.merge((image[:,:,0], image[:,:,1], image[:,:,2], mask))

leaf_with_transparent_bg[mask == 0] = [0, 0, 0, 0]

return leaf_with_transparent_bg

else:

return None

# Directories

input_folder = 'input_images/'

output_folder = 'output_images_resized/'

os.makedirs(output_folder, exist_ok=True)

# Creation Excel file

wb = openpyxl.Workbook()

ws = wb.active

ws.append(['Original_image', 'Extraction_successful'])

def process_image(image_path):

image = cv2.imread(image_path)

leaf = extract_leaf(image)

filename = os.path.basename(image_path)

output_filename = os.path.splitext(filename)[0] + '_leaf.jpg'

if leaf is not None:

# Resize to 1856 x 2784

resized_leaf = cv2.resize(leaf, (1856, 2784))

output_path = os.path.join(output_folder, output_filename)

cv2.imwrite(output_path, resized_leaf, [int(cv2.IMWRITE_JPEG_QUALITY), 40])

ws.append([filename, 'Yes'])

print(output_filename + " saved")

else:

ws.append([filename, 'No'])

print(output_filename + " not saved")

if __name__ == '__main__':

with ThreadPoolExecutor() as executor:

executor.map(process_image, glob.glob(os.path.join(input_folder, '*.JPG')))

excel_file = 'Images_info.xlsx'

wb.save(excel_file)

print("Excel file created", excel_file)

print("Process completed.")

**Supplementary Script 3.** Leaf_color_detector.py

""" @author: Matthias Pfeifer """

import os

import cv2

import numpy as np

import pandas as pd

def remove_black_background(image):

hsv = cv2.cvtColor(image, cv2.COLOR_BGR2HSV)

lower_black = np.array([0, 0, 0])

upper_black = np.array([180, 255, 30])

mask_black = cv2.inRange(hsv, lower_black, upper_black)

mask_black = cv2.bitwise_not(mask_black)

result = cv2.bitwise_and(image, image, mask=mask_black)

return result, mask_black

def calculate_percentage(mask, removed_bg_mask):

total_pixels = mask.size

bg_pixels = np.sum(removed_bg_mask == 0)

valid_pixels = total_pixels - bg_pixels

mask_pixels = np.sum(mask == 255)

percentage = (mask_pixels / valid_pixels) * 100 if valid_pixels > 0 else 0

return percentage, valid_pixels

def apply_morphological_operations(mask):

kernel = np.ones((5, 5), np.uint8)

mask_closed = cv2.morphologyEx(mask, cv2.MORPH_CLOSE, kernel)

return mask_closed

def process_image(image_path):

image = cv2.imread(image_path)

image_no_bg, removed_bg_mask = remove_black_background(image)

hsv = cv2.cvtColor(image_no_bg, cv2.COLOR_BGR2HSV)

# Adjust color range if needed

lower_green = np.array([35, 40, 40])

upper_green = np.array([85, 255, 255])

lower_yellow = np.array([20, 100, 85])

upper_yellow = np.array([30, 255, 255])

lower_brown = np.array([0, 20, 10])

upper_brown = np.array([30, 255, 170])

# Masks for green, brown, yellow

mask_green = cv2.inRange(hsv, lower_green, upper_green)

mask_brown = cv2.inRange(hsv, lower_brown, upper_brown)

mask_yellow = cv2.inRange(hsv, lower_yellow, upper_yellow)

# Adjustment for grown mask

mask_brown = apply_morphological_operations(mask_brown)

# Percentage Calculation

percentage_green, valid_pixels = calculate_percentage(mask_green, removed_bg_mask)

percentage_brown, _ = calculate_percentage(mask_brown, removed_bg_mask)

percentage_yellow, _ = calculate_percentage(mask_yellow, removed_bg_mask)

# Calculation of unassigned pixels

total_percentage = percentage_green + percentage_brown + percentage_yellow

unassigned_percentage = 100 - total_percentage

# Adjusting the values so that the sum equals 100%

adjustment_factor = (100 / total_percentage) if total_percentage > 0 else 1

adjusted_green = percentage_green * adjustment_factor

adjusted_brown = percentage_brown * adjustment_factor

adjusted_yellow = percentage_yellow * adjustment_factor

return percentage_green, percentage_yellow, percentage_brown, unassigned_percentage, adjusted_green, adjusted_yellow, adjusted_brown

# Input and output settings

input_folder = 'output_images_resized'

output_excel_path = 'output_images_color_detection.xlsx'

# Initialize results

results_data = {'Filename': [], 'Percentage Green': [], 'Percentage Yellow': [], 'Percentage Brown': [], 'Unassigned Pixels (%)': [],

'Adjusted Green (%)': [], 'Adjusted Yellow (%)': [], 'Adjusted Brown (%)': []}

for image_file in os.listdir(input_folder):

if image_file.lower().endswith('.jpg'):

image_path = os.path.join(input_folder, image_file)

# Analyze image

(percentage_green, percentage_yellow, percentage_brown,

unassigned_percentage, adjusted_green, adjusted_yellow, adjusted_brown) = process_image(image_path)

results_data['Filename'].append(image_file)

results_data['Percentage Green'].append(percentage_green)

results_data['Percentage Yellow'].append(percentage_yellow)

results_data['Percentage Brown'].append(percentage_brown)

results_data['Unassigned Pixels (%)'].append(unassigned_percentage)

results_data['Adjusted Green (%)'].append(adjusted_green)

results_data['Adjusted Yellow (%)'].append(adjusted_yellow)

results_data['Adjusted Brown (%)'].append(adjusted_brown)

# Save results to an Excel file

df_results = pd.DataFrame(results_data)

df_results.to_excel(output_excel_path, index=False)

print("Analysis results with adjustments have been saved in", output_excel_path)
